# UbasM: An effective balanced optical clearing method for intact biomedical imaging

**DOI:** 10.1038/s41598-017-12484-3

**Published:** 2017-09-22

**Authors:** Lingling Chen, Guiye Li, Yamin Li, Yingchao Li, Haiou Zhu, Li Tang, Paul French, James McGinty, Shuangchen Ruan

**Affiliations:** 1 College of Optoelectronics Engineering, Shenzhen University, Shenzhen, Guangdong, 518060 P. R. China; 2 Department of Medicine, Shenzhen University, Shenzhen, Guangdong, 518060 P. R. China; 3 Photonics Group, Department of Physics, Imperial College London, London, SW7 2AZ UK

## Abstract

Optical clearing methods can facilitate deep optical imaging in biological tissue by reducing light scattering and this has enabled accurate three-dimensional signal visualization and quantification of complex biological structures. Unfortunately, existing optical clearing approaches present a compromise between maximizing clearing capability, the preservation of fluorescent protein emission and membrane integrity and the speed of sample processing – with the latter typically requiring weeks for cm scale tissue samples. To address this challenge, we present a new, convenient, aqueous optical clearing agent, termed UbasM: Urea-Based Amino-Sugar Mixture, that rapidly renders fixed tissue samples highly transparent and reliably preserves emission from fluorescent proteins and lipophilic dyes in membrane integrity preserved tissues. UbasM is simple, inexpensive, reproducible and compatible with all labeling methods that we have encountered. It can enable convenient, volumetric imaging of tissue up to the scale of whole adult mouse organs and should be useful for a wide range of light microscopy and tomography techniques applied to biomedical research, especially the study on organism-level systems biology at multiple levels.

## Introduction

Biomedical research relies extensively on optical imaging and measurement techniques with an increasing emphasis on quantitative three-dimensional (3D) imaging, for which a range of new optical imaging techniques^1–9^ and novel labeling techniques^10–12^, offer exciting prospects for comprehensive studies of biological structure and function. Today, much biomedical research is being translated from studies of cell monolayers on glass or plastic to *in situ* measurements in organs or even whole animals and there is increasing demand for techniques to provide molecular contrast with high (cellular) resolution in thick tissue samples. This demand is essential for system-level identification and quantitative analysis and would be invaluable for profound understanding in organism-level biology systems at multiple levels, e.g. 3D structure-function relationship at the whole-brain level.

Conventionally, high-resolution 3D imaging of relatively large tissue samples would be realized using mechanical sectioning techniques combined with staining to provide optical contrast^13–16^. However, such methods require expensive equipment, are labor intensive and require sophisticated data registration and, since fine detail could be lost during sample processing, can fail to faithfully reconstruct 3D volumes. In recent decades a variety of 3D “mesoscopic” (mm-cm) optical imaging techniques have been developed, including light sheet microscopy^6–8^ (e.g. selective plane illumination microscopy (SPIM) and ultramicroscopy), optical projection tomography (OPT)^9^ and scanning laser optical tomography (SLOT)^17^. These provide a potentially cost-effective and relatively rapid means to acquire high-resolution 3D images of intact samples with fluorescence and/or absorption contrast. This is particularly powerful when combined with labelling techniques based on genetically expressed fluorescent proteins (FP). Such techniques are being applied to facilitate system-level studies, such as mapping neural circuits in the brain^18–21^ and cellular behavior in developing embryos^22,23^.

However, the utility of optical imaging in such studies is limited by the absorption and scattering of biological tissue, which limits imaging with subcellular resolution to a few hundred micrometers of the surface for most biological tissues, even when using two-photon microscopy^5^. *In vivo* optical imaging is practical in some inherently transparent small organisms (e.g. *D. rerio* and *C. elegans*) but 3D volumetric imaging of larger, less transparent organisms/tissues (e.g. mouse brain, pancreas, lung, etc.) requires optical clearing methods to render intact tissue samples optically transparent by reducing light scattering and minimizing absorption through the optimization of the imaging radiation wavelength.

Visible and infrared radiation is scattered by variations of refractive index (RI) in a sample. To reduce optical scattering and thereby enhance the ability to visualize internal structures in tissue, the internal RI variations can be reduced by optical clearing techniques that essentially replace water in a sample with higher refractive index materials in order to match the RI of other tissue components and approach a uniform RI throughout the sample. In early studies^6,24^, an organic solvent-based clearing reagent known as “Murray’s Clear solution” or BABB (a 1:2 mixture of benzyl alcohol and benzyl benzoate) was employed to clear tissue samples based on its hydrophobicity and high RI (~1.56). Subsequently, many new optical clearing techniques have been reported that utilize a range of different materials to replace the water initially present in the sample. A selection of these approaches and their relative advantages are summarized in Table 1. The first category, starting with BABB, are organic solvent based methods, that are relatively fast when clearing larger samples and achieve good transparency but tend to suppress emission from fluorescent proteins and compromise cell membrane integrity (MI). Of these, 3DISCO^25^ has been reported to achieve a remarkably high level of transparency within a few days for different types of tissues (e.g. mouse lung, spleen and brain). However, these reagents rapidly quench FP emission as the organic chemicals denature the proteins. To address this severe limitation, other organic agents, such as dibenzyl ether, methyl salicylate, 2, 2′-thiodiethanol and tetrahydrofuran^26–28^, and a modification of BABB using 1-propanol or tert-butanol during dehydration^29^ have been proposed to mitigate the FP quenching and been shown to perform better, but some FPs (e.g. YFP) are still quenched. uDISCO^30^, a modified version of 3DISCO, was recently reported to be capable of preserving FP emission.

**Table 1:** Comparison of UbasM properties with other optical clearing methods. The table summarizes general properties and performance indicators for UbasM and other major published clearing methods. These methods are categorized mainly into aqueous solution-/organic solvent-based and hydrogel embedding methods. To evaluate clearing efficiency, mm-thick adult mouse brain slice and 4-week old mouse hemisphere were used as small (~mm) and large samples (~cm). SeeDB was only demonstrated to embryos and young mice and the resulting sample transparency is insufficient for one-photon imaging of the adult organ. The complex uDISCO processing (i.e. 8–9 steps) required at least 15 hours^30^. Conclusions were evaluated from experiments performed in this paper (indicated by bold text) and also based on published information. (RT – room temperature; N.T. – not tested)

Method (Ref)	Agents		Clearing Performance					Handling	Incubation Temp.
	Main (Detergent)	Clearing Mode (steps)	Capability	Efficiency		FP preservation	DiI compatibility		
				Slice (~mm)	Hemisphere (~cm)				
**Organic solvent-based methods**									
**BABB** (ref.^24^)	Benzyl alcohol Benzyl Benzoate	Replacing water with organic solvents (5–6 steps)	**Very Strong**	**Slow >4.5 hr**	**Very Fast 3–7 d**	**Very poor**	**Poor**	**Needs Care**	**RT**
**3DISCO** (ref.^25^)	Benzyl Ether	Replacing water with organic solvents (6 steps)	**Very Strong**	**Slow >3.5 hr**	**Very Fast 3–7 d**	**Poor**	**Poor**	**Needs Care**	**RT**
iDISCO (ref.^40^)	Benzyl Ether	Replacing water with organic solvents (6 steps)	Very Strong	Slow >3.5 hrr	Very Fast 3–7 d	Poor	Poor	Needs Care	RT
**uDISCO** (ref.^30^)	BABB(4): DBE(1)	Replacing water with organic solvents (8–9 steps)	**Very Strong**	**Very Slow>15 hr**	**Very Fast 3–7 d**	**Medium**	**Poor**	**Needs Care**	**> 26 °C**
**Aqueous solution-based methods**									
**Scale** (ref.^31^)	Urea Glycerol (0.1%Triton X-100)	Water-based chemical agents (1–2 steps)	**Medium**	**Very Slow >15 hr**	**Very Slow months**	**Excellent**	**N.T.**	**Very Easy**	**RT**
**SeeDB** (ref.^34^)	Fructose	Sugar solutions (6 steps)	**Weak**	**Slow >2 hr**	—	**Excellent**	**Good**	**Easy (viscous)**	**37 °C**
**CUBIC** (ref.^32^)	Urea Amino-alcohol (Triton X-100 15%, Reagent-1 0.1%, Reagent-2)	Detergent-based chemical agents (3 steps)	**Strong**	**Fast 1–2 hr**	**Fast 7–12 d**	**Excellent**	**Poor**	**Very Easy**	**RT (or 37 °C)**
**ScaleS** (ref.^33^)	Urea Sorbitol (0.2% Triton X-100)	Water-based chemical agents (5 steps)	**Strong**	**Medium >2 hr**	**Medium 2–4 weeks**	**Excellent**	**Good (Poor for SQ(5))**	**Very Easy**	**37 °C**
**UbasM**	Urea Amino-sugar (0.2% Triton X-100)	Water-based chemical agents (3 steps)	**Strong**	**Fast 1–2 hr**	**Fast 7–12 d**	**Excellent**	**Good**	**V** **ery Easy**	**RT (or 30 °C)**
**Hydrogel embedding methods**									
CLARITY (ref.^37^)	SDS Boric acid (SDS 4%)	Hydrogel embedding +removal of lipids with electrophoresis	Very Strong	- N.T.	Medium 2–4 weeks	N.T.	N.T.	Complicated Needs device Careful	37 °C
PACT (ref.^38^)	SDS Histodenz (SDS 8%)	Hydrogel embedding + removal of lipids with 8% SDS	Very Strong	- N.T.	Medium 2–4 weeks	Medium	Poor	Careful	37 °C

In order to develop a less severe optical clearing approach with reduced quenching of FP emission and less degradation of MI, a number of water-based chemical agents have been developed^31–35^. Sca*l*e^31^, a urea-based reagent, was used to clear tissue such as brain samples, achieving moderate optical transparency with excellent retention of FP emission. This approach is relatively slow (requiring weeks to months to clear cm scale tissue samples) and produces some volume enlargement. The latter may be acceptable for most experiments—and indeed, this phenomenon can be exploited in the technique of “expansion microscopy”^36^ to enhance resolution. CUBIC^32^, a mixture of urea, amino-alcohol and detergent, achieved a high level of optical transparency in a few days but compromised MI due to the high concentration of lipid-extracting detergent (15% Triton X-100)^33^. Sca*l*eS^33^, a mixture of urea and sorbitol, was recently reported to be capable of preserving detailed structures by using a very low concentration of detergent (0.2% Triton X-100) and achieving reasonable transparency after several days. Alternatively, a fructose-based reagent without detergents, SeeDB^34^, provided clearing of brain samples within days with no tissue deformation, but this simple hydrophilic reagent achieved relatively low optical transparency. FRUIT^35^, a modified version of SeeDB mixing fructose and urea, improved permeability and accelerated clearance but still achieved low transparency. However, they were sufficient to permit two-photon imaging.

A third category of clearing approaches involves the transformation of intact tissue into a nanoporous hydrogel-hybridization form. For the method termed CLARITY^37^ optical clearing is achieved by aggressively removing lipids using electrophoresis to provide high transparency of cm scale tissue samples within a few weeks. PACT^38^, a clearing protocol derived from CLARITY, uses 8% SDS (i.e. ionic detergents) to extract lipids from tissue-hydrogel matrix to clear tissue slices and rodent whole organs, but with similar concerns regarding the degradation of MI. While these approaches produce impressive results, they entail the use of relatively complex apparatus and have been found to compromise MI^33^.

Although previous methods each have advantages, none has yet been reported that provide rapid clearing to high transparency while preserving FP emission and membrane integrity. In particular, achieving both rapid effective clearing and FP/MI preservation has presented a significant challenge to date. We believe that the new method presented here, UbasM (Urea-Based Amino-Sugar Mixture), offers unprecedented performance in terms of convenience, clearing rate and satisfactory FP emission/membrane integrity preservation while achieving sufficient transparency to permit 3D imaging of cm scale samples with single-photon excited fluorescence. Here we demonstrate its performance for 3D optical imaging of samples labelled with a range of fluorophores, including various FPs and lipophilic dyes, allowing imaging of fixed tissue samples with single-photon-excitation light microscopy at cellular resolution. We illustrate its utility with 3D imaging of metastatic cells of murine breast cancer and melanoma cancer cell lines from subcutaneous/foot pad locations in whole-organ preparations from a mouse model and of amyloid-β (Aβ) in aged and diseased brain samples from an Alzheimer’s disease (AD) mouse model. Together, our findings suggest that UbasM is a simple, rapid, efficient, inexpensive, reproducible and well-balanced optical clearing method which can enable diverse applications in biomedical research and can facilitate comprehensive and quantitative analysis of cells or even functional information in organs and whole organism for further understanding of the activity of cells and cellular networks at the whole-organ level.

## Results

### A new optical clearing agent for tissue

In developing UbasM, we considered how to optimize transparency, efficiency, FP preservation, structural integrity, ease of operation and reproducibility. Because urea-based aqueous agents (e.g. Sca*l*e, CUBIC and Sca*l*eS) have the potential to fulfill these criteria, we searched for chemical compounds that can be combined with urea to improve clearing performance. After screening a number of hydrophilic small molecules, sugars and amino sugars and meglumine were found to be most effective in clearing. 1,3-Dimethyl-2-imidazolidinone is a hydrophilic small molecule that was found to enhance tissue-clearing. Meglumine, an amino sugar in which the cationic amino can contribute to solvating anionic lipids in the tissue, aids lipid removal and tissue dehydration. Detergents can also be effective agents to remove lipids and therefore Triton X-100 is included in UbasM, although at lower concentration (0.2%) compared to CUBIC (15% Triton X-100 in Reagent-1) in order to reduce the degradation of MI. Our first reagent, which we refer to as Ub-1 (RI ~1.45), provides a high level of transparency when applied to brain slices, hemispheres and other organs, but also results in a size expansion (~120–160%). To further improve the clearing performance, a second reagent, Ub-2, which includes urea, sucrose and 1,3-Dimethyl-2-imidazolidinone, can be applied. This has a RI in the range 1.47–1.48, providing a better match to the tissue RI and it recovers the original tissue volume after washing the treated tissue in buffer (i.e. phosphate-buffered saline, PBS).

### Optical clearing of biological tissue

**Figure 1:**
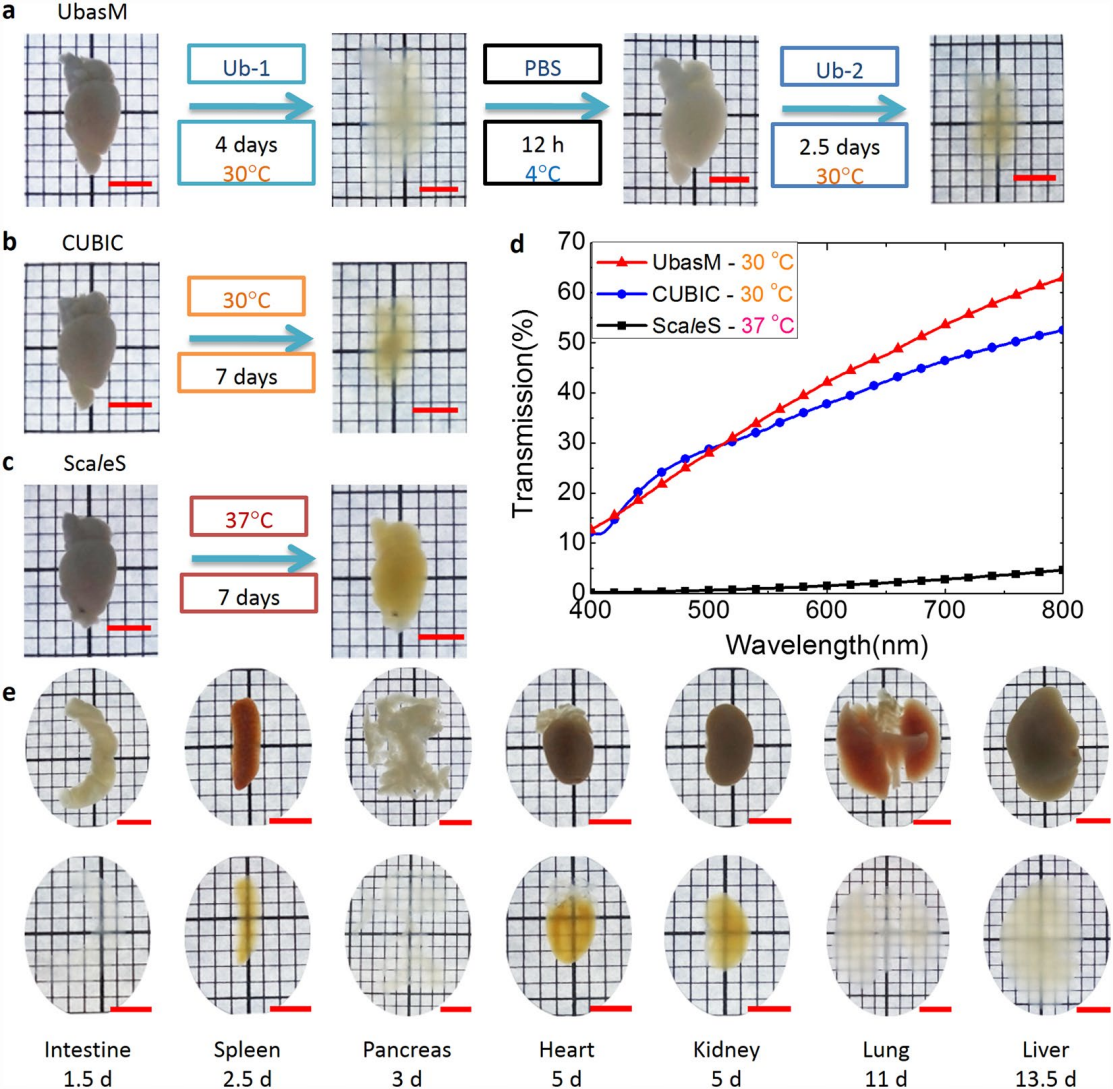
Optical clearing of the whole-organ using UbasM. (**a**) Procedure of the UbasM clearing protocol and hemisphere brain samples cleared with UbasM at 30 °C for 7 days, (**b**) hemisphere brain samples cleared with CUBIC at 30 °C for 7 days and (**c**) hemisphere brain samples cleared with Sca*l*eS at 37 °C for 7 days. All hemisphere brain samples were from C57/BL 4-week-old mice. (**d**) Transmittance curves of mouse brain samples cleared with various clearing agents after 7 days as shown in (a-c). (**e**) Clearing performance of whole organs (from C57/BL 8-week-old female mouse) at 30 °C for 3~10 days (clearing time depends on tissue). Organs such as intestine, spleen and pancreas tend to become transparent more quickly. Scale bars, 5 mm.

The overall clearing protocol entails a sequential incubation of fixed samples in Ub-1, PBS and then Ub-2, as shown in Supplementary Fig. 1. To illustrate the practical performance of UbasM for whole-organ imaging, Fig. 1 shows the clearing achieved for various whole organs (e.g. heart, lung, kidney, pancreas, *etc*). A high degree of transparency was obtained within a few days (~3–10 days depending on the organ) with minimal volume variation (~95–115%) after the expansion and contraction phases of the clearing procedure. To clear ~cm scale tissue samples, such as a mouse brain hemisphere, requires ~7 days, which is comparable to CUBIC (~9 days, Supplementary Fig. 2) and faster than Sca*l*eS (~weeks, Supplementary Fig. 2).

To evaluate the tissue transparency achieved, light transmittance was quantified in 4-week old mouse hemisphere samples. After 7 days, hemispheres treated with UbasM were slightly more transparent than those treated with CUBIC and significantly more transparent than those cleared with Sca*l*eS (Fig. 1a–d). Treating these samples with Sca*l*eS for 28 days yielded similar transparency to that achieved after 7 days with UbasM (Supplementary Fig. 2). We note that Sca*l*eS requires an incubation temperature of 37 °C to prevent precipitation of crystals while UbasM and CUBIC can be performed at 30 °C or even at room temperature (20 °C) which is convenient for experiments, although clearing is faster and can be more effective at higher temperatures. While applying UbasM and CUBIC to tissue at 37 °C resulted in a higher level of transparency, this also caused the treated samples to become soft and fragile while the samples treated at lower incubation temperatures (30 °C or 20 °C) did not show marked fragility. Considering that the slightly higher level of transparency obtained at 37 °C could also be achieved at lower temperatures (e.g. 30 °C) by increasing the incubation time at each step (Supplementary Fig. 2), the incubation temperature for the application of UbasM was kept below 30 °C.

### Preservation of FP emission

**Figure 2:**
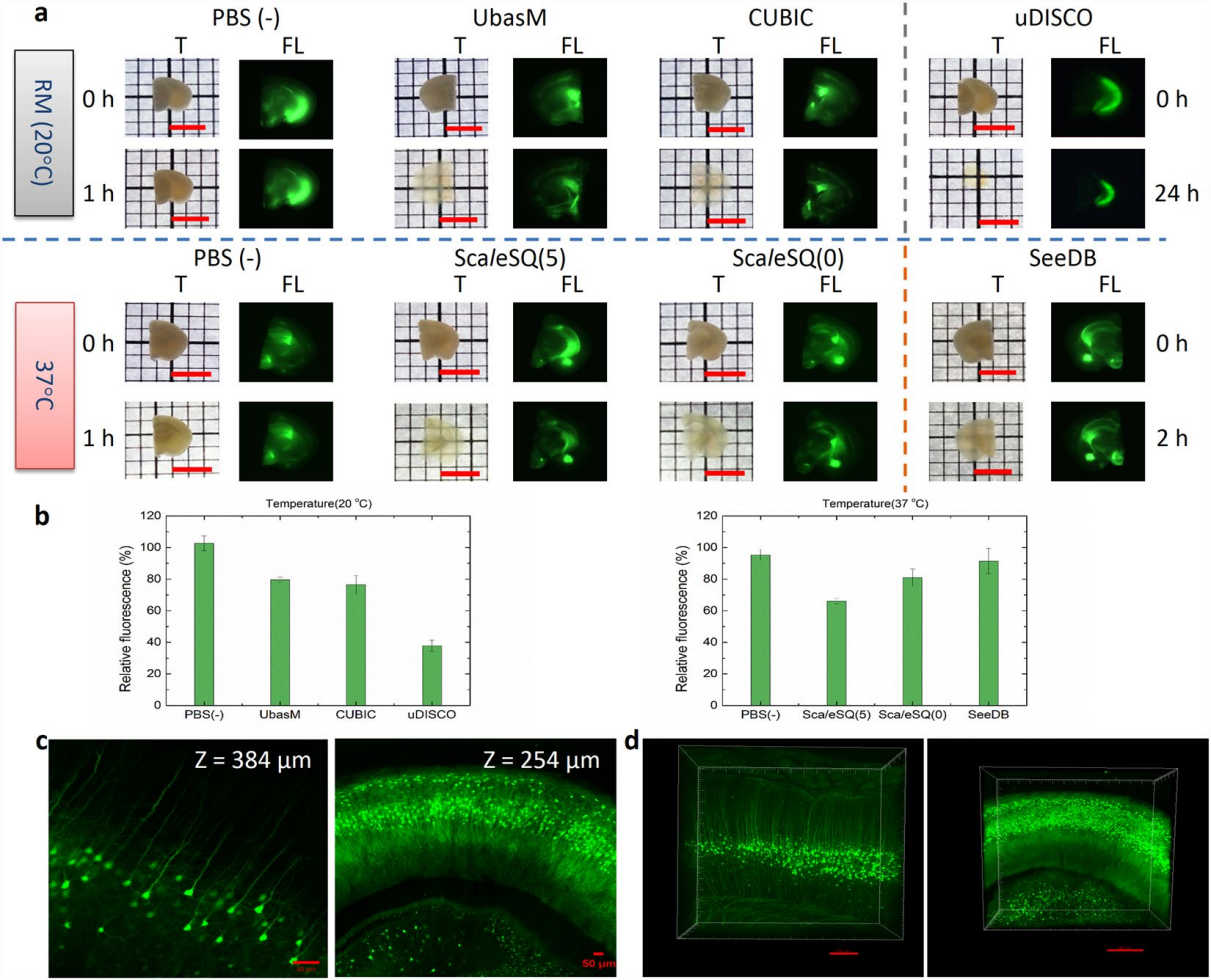
Comparison of optical transparency and fluorescence protein preservation. (**a**) Representative images for the evaluation of UbasM, CUBIC and uDISCO (incubation at room temperature 20 °C) and Sca*l*eSQ(5), Sca*l*eSQ(0) and SeeDB (incubation at 37 °C) and the control (PBS(-)) by using 1-mm-thick brain slices from 11-week-old Thy1-YFP (H-line) mice. In each method, transmission images are shown left and fluorescence images shown right. The incubation time for UbasM, CUBIC, Sca*l*eSQ(5) and Sca*l*eSQ(0) was set 1 h, for SeeDB 2 h while for uDISCO 24 h. (**b**) The YFP preservation ratio for UbasM, CUBIC and uDISCO (at 20 °C) and Sca*l*eSQ(5), Sca*l*eSQ(0) and SeeDB (at 37 °C). Comparisons were made using at least three Thy1-YFP (H-line) mice for each method. (**c**) Confocal section images of YFP-expressing neurons in UbasM-treated brain slices of a Thy1-YFP (H-line) mouse (11-week-old, male) in cortex (Z = 384 µm) and hippocampus (Z = 254 µm) region. (**d**) 3D rendering of YFP-expressing neurons in cortex and hippocampus region. Images were taken by single-photon excitation confocal microscopy (Zeiss, LSM 710). Scale bars represent 5 mm (**a**), 50 µm (**c**) and 200 µm (**d**).

To examine preservation of FP emission after clearing, UbasM and other clearing techniques were applied to multiple 1–2 mm thick Thyl-YFP (H-line)^39^ mouse brain slice samples, in which yellow fluorescence protein (YFP) is expressed at high levels in motor and sensory neurons, as well as subsets of central neurons. These samples were fixed and treated with UbasM, CUBIC and uDISCO with incubation at room temperature (~20 °C) or with Sca*l*eS, SeeDB and the control PBS(-) incubated at 37 °C. The resulting cleared brain slices were characterized for comparative light transmission and YFP fluorescence as shown in Fig. 2a. Although clearing with the organic solution uDISCO produced high transparency, we observed significant quenching of the YFP signal and also significant volume reduction compared to the aqueous agents (i.e. CUBIC, Sca*l*eS, SeeDB and UbasM) as shown in Fig. 2b. Furthermore, the more complex uDISCO processing protocol (i.e. 8–9 steps) required at least 15 hours^30^, compared to ~1–2 hours for the other techniques. In contrast, SeeDB exhibited the best preservation of FP emission, but this relatively simple hydrophilic reagent resulted in the lowest optical transparency after 2 hours treatment. The Sca*l*eS-treated samples preserved YFP fluorescence but were less transparent than those cleared with UbasM and CUBIC, and especially those treated with non-detergent Sca*l*eSQ(0). CUBIC exhibited comparable capabilities compared to UbasM in terms of FP preservation and clearing efficiency.

These results exhibit the trade-off between clearing transparency and FP preservation for the different clearing methods and verify that UbasM provides rapid clearing to achieve a high level of transparency without sacrificing FP fluorescence. For experiments requiring tissue thickness of 1 mm or less, such as high throughput mapping of serial brain sections, UbasM and CUBIC provide rapid and effective clearing with ease of practical implementation. To demonstrate the FP imaging performance, YFP-expressing neurons in 1 mm-thick UbasM-treated brain slices were imaged by single-photon excitation confocal laser scanning microscopy (CLSM). The sectioned YFP fluorescence images and 3D reconstructions of cortex and hippocampus are shown in Fig. 2c,d and Videos 1,2.

### Preservation of membrane integrity and compatibility with lipophilic fluorescent dyes

**Figure 3:**
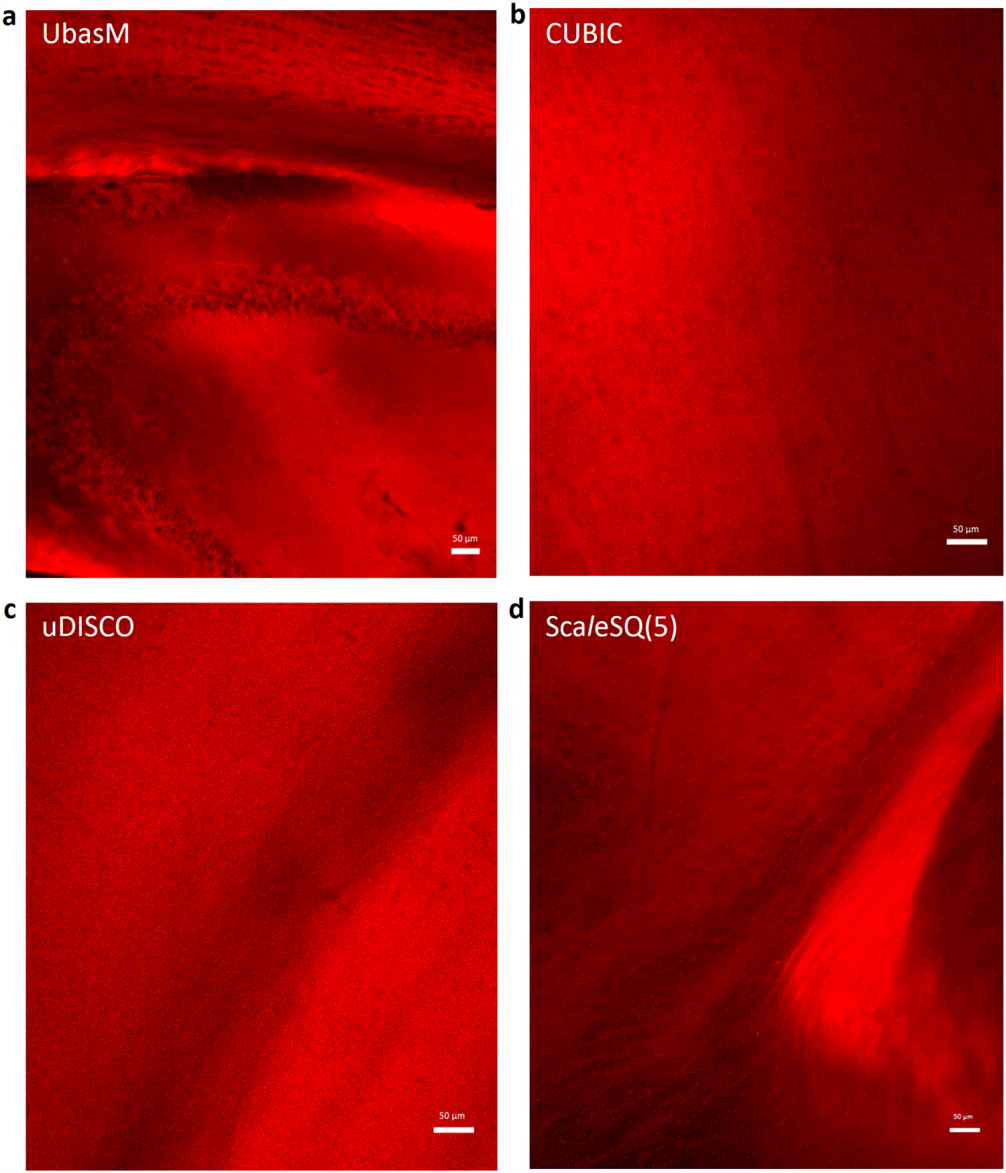
Compatibility of UbasM and current advanced clearing methods for lipophilic fluorescent dye - DiI. (**a**–**d**) Confocal fluorescence images of DiI-labeled neural tracts in the hippocampus (in the 1-mm-thick brain slices) with optical clarification by UbasM, CUBIC, uDISCO and Sca*l*eSQ(5) respectively. Significant deformation was noted after CUBIC, uDISCO treatment and obvious deformation after Sca*l*eSQ(5) treatment while no deformation after UbasM treatment. Scale bars: 50 μm. More detailed results were obtained before, after 1 h and 24 h clarification (Supplementary Fig. 4).

Membrane staining with lipophilic fluorescent dyes, especially DiI, is the primary method for tracing neuronal tracts in brain samples and is only effective if the membrane integrity is preserved. However, the current rapid and efficient clearing methods, such as CUBIC, Sca*l*eSQ(5) and uDISCO are not compatible with DiI due to the high concentration of membrane-damaging detergent (15% in CUBIC, 5% in Sca*l*eSQ(5)) or organic solvents (uDISCO) that extract lipids during the aggressive clearing. Figure 3b–d illustrates the resulting loss of detail of the MI, especially verifying the severe degradation of structure integrity introduced by CUBIC and uDISCO clearing, which agrees with the results presented in ref.^33^. Because of the low (0.2%) level of Triton X-100 in UbasM, it was found to be compatible with DiI staining since the plasma membranes remain intact, as with other mild slow-clearing methods (e.g. SeeDB, FRUIT and Sca*l*eSQ(0)). This advantage is of crucial importance since lipophilic dyes are the only choice for fluorescent neuronal tracing of post-fixed brains. Thus, UbasM is shown to permit immunolabeling of intact membranes while providing a rapid clearing and a high level of transparency.

### Rapid high-performance 3D volumetric imaging

**Figure 4:**
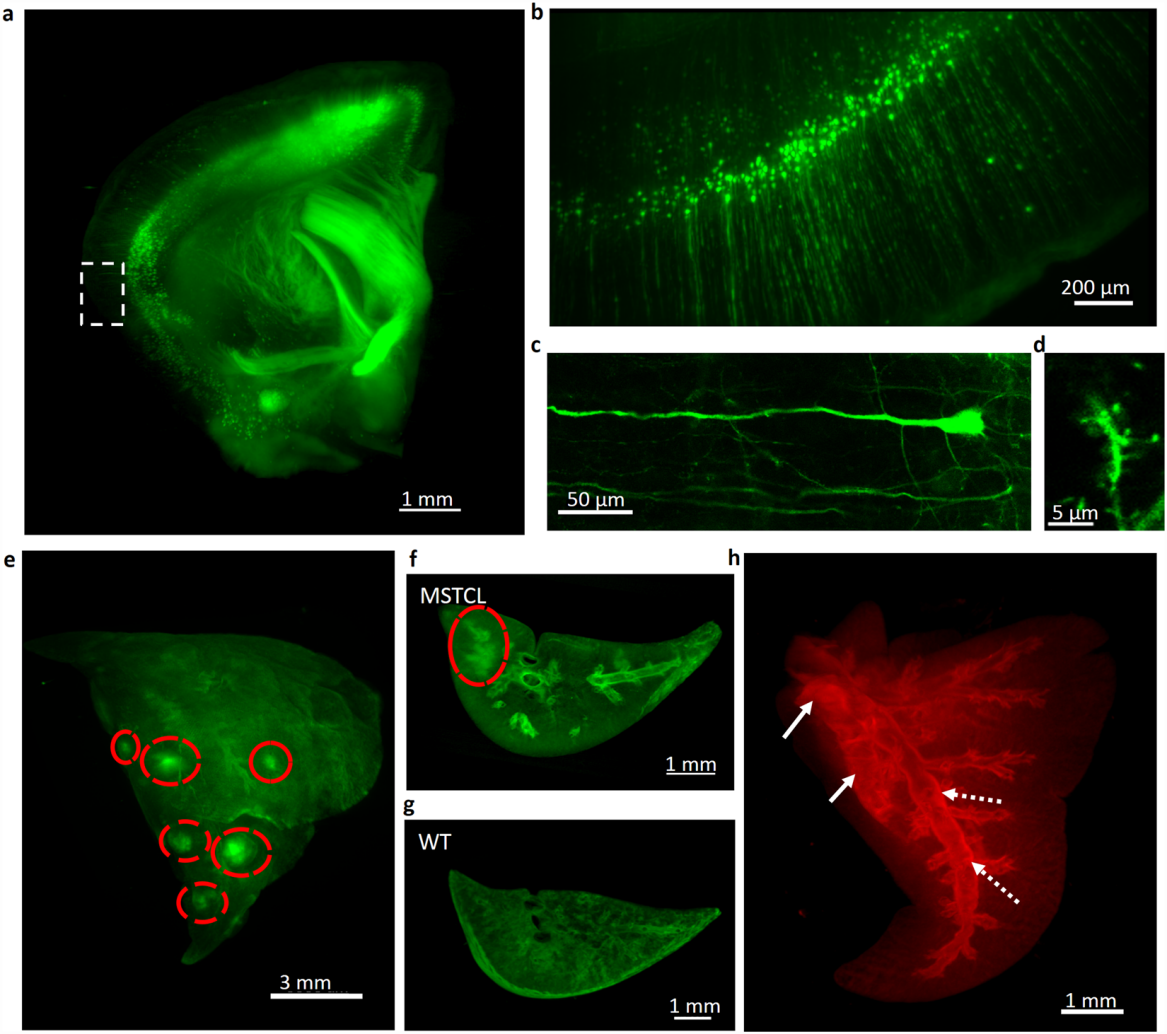
Rapid and high-performance 3D volume imaging of various samples clarified by UbasM. (**a**) A 3D reconstruction fluorescent image of thy1-YFP (H-line) mouse brain slice (10-week-old female) acquired with OPT. Magnified view of the boxed in (**a**) is shown in (**b**) acquired with OpenSPIM. After UbasM clearing, high-magnification imaging of fine structures about a single neuron (**c**) and dendritic spines (**d**) with confocal microscopy. 3D reconstruction fluorescent images of (**e**) a mouse half-lung sample and (**f**) a tip of lung sample showing metastases of murine breast cancer cell line 4T1-ZsGreen1 from foot pad locations acquired with OPT (red circles indicate the clusters of metastasized cells). (**g**) 3D reconstruction fluorescent images of a tip of the control lung sample (from wild type mouse) showing the autofluorescent structures as comparison. (**h**) 3D reconstruction images of fluorescent metastatic cancer cells (i.e. melanoma cell line B16F10-tdTomato from subcutaneous locations) within a mouse lung sample acquired with OPT. The comparison of cancer model and the wild type is shown in Supplementary Fig. 7. It is noted that there is bright fluorescence of metastatic cancer cells within the bronchi that may indicate the paths of cancer cells spreading through the lymphatic or vascular circulation (indicated by white dash arrows). The bright fluorescent areas (indicated by white solid arrows) show where the malignancy has infiltrated the lung tissue. (**a**,**b**), (**e**–**h**) are maximum intensity projections while (**c**,**d**) are confocal section images. WT – wild type; MSTCL - metastatic spread of tumor cell line. Scale bars represent 1 mm (a), 200 µm (**b**), 50 µm (**c**), 5 µm (**d**), 3 mm (**e**), 1 mm (**f**,**g**,**h**).

We demonstrate the utility of the UbasM clearing method by combining it with laser scanning confocal fluorescence microscopy, light sheet microscopy and OPT for 3D imaging of mm-scale tissue blocks and intact whole-organ samples. A transgenic mouse, thy1-YFP (H-line), was used as a model for typical large-tissue-block brain imaging. Figure 4a shows the 3-D YFP image of a 2-mm-thick brain slice from this model cleared using UbasM and imaged using OPT, Fig. 4b represents a higher resolution 3D image acquired using an OpenSPIM^40^ system and Fig. 4c,d show higher resolution confocal fluorescence microscopy images of the same sample. Supplementary Fig. 5 shows OPT images of a 4 mm thick slice of brain tissue from the thy1-YFP (H-line) transgenic mouse and Video 3 shows a rendering of the 3D reconstruction of the OPT data. To demonstrate whole organ imaging, a stably transfected murine breast cancer cell line 4T1-ZsGreen1 and a melanoma cell line B16F10-tdTomato were both introduced into female BALB/c mice and C57BL/6 mice respectively to provide models of cancer where intact whole-organ clearing and volumetric imaging can be applied to investigate metastasis of these tumor cell lines from subcutaneous/foot pad locations. Exemplar 3D images reconstructed from OPT data are shown in Fig. 4e–g, Videos 4 & 5 and Supplementary Figs 6, 7. These reconstructed images of the cleared mouse brain, lung and liver samples demonstrate the potential 3D visualization and localization of spatial expression patterns and the study of structural detail achievable using FPs (including ZsGreen1, YFP and tdTomato) with UbasM.

### 3D immunohistochemistry of Aβ plaques in adult mouse brain using UbasM

**Figure 5:**
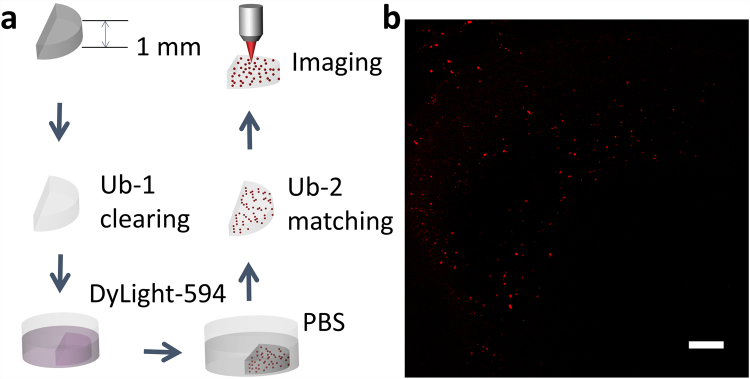
Imaging of Aβ plaques by using modified UbasM protocol. (**a**) Schematic diagrams illustrating the procedure of clarification, Aβ plaques (red dots) immunostaining and imaging. (**b**) 3D reconstructed image (maximum intensity projection) of Aβ plaques (red dots) in a 15-month-old AD mouse brain slice. Scale bar: 100 μm.

Immunolabeling is a powerful tool for biological research and medical diagnosis. In order to map molecular composition and morphology of biomedical specimens, it is usually necessary to mechanically section tissue before staining. However, deep tissue immunolabeling has recently been demonstrated with organic-solution clearing methods, such as iDISCO^41^, and various aqueous clearing agents have been shown to enable 3D imaging of immunostained brain samples because of the high permeability^32,33^. The high permeability of the UbasM reagents conferred by the high concentration of urea enables tissue slices to be effectively cleared and immunostained using the protocol illustrated in Fig. 5a. We applied this procedure to image 20 brain slices taken from 3 AD mice and 3 wild type control mice. To obtain the images shown in Fig. 5b, Ub-1 was applied to brain tissue (1 mm-thick slice) from a 15-month-old AD mouse, and also to a wild-type (WT) mouse as a control. Following this clearing stage, the brain slice was incubated with fluorescent antibodies (DyLight594) to label Aβ plaques and after this immunostaining stage, the tissue was treated with Ub-2 for enhanced RI matching. The resulting cleared, stained samples were imaged with a single-photon-excitation CLSM, with which immunostained signals were detectable at depths greater than 600 µm (shown in Supplementary Fig. 8). The uniform penetration of DyLight594 into UbasM-treated samples enabled quantitative labeling of Aβ plaques inside these adult brain slices. These initial results (also shown in Supplementary Figs. 8, 9) are in agreement with previous observations^33^.

## Discussion

We have demonstrated that UbasM is a straightforward, rapid, effective and scalable tissue optical clearing method for high-resolution 3D imaging at multiple spatial scales (microscopic/mesoscopic/macroscopic). Compared with current optical clearing methods, our findings suggest UbasM presents the best balance reported to date between clearing performance and preservation of FP emission and membrane integrity, as summarized in Table 1.

We note that when optical clearing is applied to adult animal tissues, the efficacy of clearing techniques can be reduced by the accretion of impermeant tissue barriers. The Sca*l*e and SeeDB clearing methods have mainly been applied to embryos and young mice since the cleared sample transparency is insufficient for single-photon imaging of adult organs. Conversely, BABB, 3DISCO, iDISCO provide rapid robust optical clearing of adult tissue samples but do not preserve FP emission. CUBIC and uDISCO do provide effective clearing and preserve FP emission but unfortunately are not compatible with DiI staining since the aggressive lipid extraction (using high concentrations of detergents or organic solvents) can compromise membrane integrity and potentially other tissue ultrastructures. In contrast, Sca*l*eS and UbasM, which both contain ≤ 0.2% Triton X-100, cause less degradation of MI and are compatible with DiI staining. This compatibility with lipophilic fluorescent dyes for membrane staining is a notable feature because it means such mild clearing methods could be applied to study neuronal circuits in non-transgenic experiment systems, including *ex vivo* human brain samples. However, Sca*l*eS does not clarify tissues to the same degree as UbasM and the clearing rate is at least 50% lower. The more rapid Sca*l*eSQ(5) formulation applied to brain slices does not match the clearing performance of just the Ub-1 reagent in terms of transparency and clearing rate and can lead to partial loss of MI as shown in ref.^33^ and also indicated by our DiI staining results.

In terms of practical convenience, UbasM simply requires the immersion of tissue samples in reagents Ub-1 and Ub-2. No specialized equipment is required, which makes the technique widely accessible and suitable for the efficient clearing of large numbers of samples. While current water-based clearing methods (e.g. SeeDB, Sca*l*e, CUBIC, Sca*l*eS) also have simple protocols, UbasM provides superior performance in terms of clearing speed and/or compatibility with DiI staining. UbasM is also relatively insensitive to incubation temperature. The UbasM processing reported here was carried out at room temperature or 30 °C and the treated samples were less fragile compared with those processed with other water-based reagent-cleared samples (e.g. CUBIC, Sca*l*eS). This could be important for the dissection of larger-scale samples for 3D reconstruction or mapping.

Rapid precise whole-organ high-resolution 3D imaging requires the combination of well-balanced optical clearing with appropriate imaging techniques. Our OPT set-up typically acquires 3D image data of large tissue block or even whole-organ samples (mesoscopic/macroscopic) within a few minutes and yields 3D reconstructions with an isotropic resolution of ~9–25 µm (depending on sample size). The OpenSPIM system delivers cellular resolution (~1.3 µm), but with the accumulation of huge (~ TB) data sets acquired over typical acquisition times of a few hours for cm × cm × mm scale samples. More sophisticated LSM approaches (such as both-sided^42,43^, two-photon^44^, line-scanning, Bessel beam^45^, lattice light-sheet^46^) can provide faster imaging and superior resolution but the resulting data sets still present significant challenges in terms of data management and image processing. CLSM of mm scale sample volumes is typically prohibitively slow and results in significant photobleaching so it is more usefully restricted to regions of interest within such samples.

We believe that optical clearing with UbasM combined with 3D fluorescence imaging techniques can provide a convenient and broadly applicable platform for tissue slice and whole-organ imaging and facilitate the mapping and quantitative analysis of functional information for biomedical and other research fields.

## Materials and methods

### The UbasM protocols

UbasM-1 (Ub-1) was prepared as a mixture of 25 wt% Meglumine (Shanghai Macklin Biochemical Co., M813277), 25 wt% Urea (Sigma-Aldrich, 15604), 20 wt% 1,3-Dimethyl-2-imidazolidinone (Sigma-Aldrich, cat. no. 40727), and 0.2 wt% Triton X-100 (Sigma-Aldrich, 10789704001). UbasM-2 (Ub-2) was prepared as a mixture of 25 wt% Urea (Sigma-Aldrich, 15604), 20 wt% 1,3-Dimethyl-2-imidazolidinone (Sigma-Aldrich, 40727), and 40 wt% Sucrose (Sigma-Aldrich, S9378). For brain slice clearing, each fixed slice was immersed in 5–10 ml of Ub-1 at room temperature for 1–2 hours. For whole organ clearing, each fixed sample was immersed in 10–20 ml of Ub-1 at 30 °C for 3–5 days with gentle shaking. The treated sample was then washed with PBS several times and then stored at 4 °C for 12 hours. Then the washed sample was immersed into in 10–20 ml of Ub-2 at 30 °C for 3–5 days. After imaging, the sample was again washed with PBS, immersed in 20% (w/v) sucrose in PBS, and stocked in O.C.T. compound at –80 °C. Agarose-embedded samples typically required longer incubation times for efficient clearing. We recommend embedding the samples in agarose after Ub-1 clearing and PBS washing and minimizing the size to allow efficient penetration of Ub-2. Penetration efficiency can also be improved by clearing tissues at 37 °C but with larger size expansion, less firmness and partial quenching of fluorescent proteins.

### Mouse and sample preparation

Wild-type C57BL/6 J Mice (from Guangdong Medical Laboratory Animal Center, Foshan, China) and Thyl-YFP (H-line) mice (from Peking University Shenzhen Graduate School, ShenZhen, China) in C57BL/6 J background were used for optical clearing and imaging. 3xTg-AD mice (from College of Life Sciences and Oceanography, Shenzhen University, Shenzhen, China), which express the mutant human APPswe and tauP301L genes and the mutant mouse PS1M146V gene, were used for Aβ plaques labeling. Adult mice (4–80 weeks old) were deeply anesthetized with a mixture of 4% Chloral hydrate (Shanghai Macklin Biochemical Co., C804539)/normal saline Sodium chloride (Shanghai Macklin Biochemical Co., S805277) (wt/vol), killed by intracardiac perfusion with PBS and followed by perfusion with 4% (wt/vol) paraformaldehyde (Sigma-Aldrich, 16005) (PFA)/PBS(-) for fixation. Then the organs (e.g. brain, lung, kidney and liver) were extracted carefully and subjected to post-fixation in 4% PFA/PBS(-) at 4 °C overnight (up to 1 d).

### DiI labeling

Excised mouse brain samples (12-week-old female C57BL/6 J mice) were fixed with 4% PFA in PBS at 4 °C overnight. DiI labeling was performed as described as ref.^34^. A small incision was made and a small DiI C18 (3) crystal was placed into the incision. The brain sample was then incubated in 2% PFA in PBS at 37 °C for 16 days. The samples were cleared with UbasM, CUBIC, uDISCO, Sca*l*eSQ(5) and Sca*l*eSQ(0) and then imaged by using an inverted confocal microscope (Zeiss, LSM710).

### Antibodies for Aβ immunostaining

The following primary antibody (Ab) was used: mouse polyAb to Amyloid beta (Biolegend, 803002, 1:100). The following secondary Ab was used: goat polyAb to mouse IgG conjugated to DyLight 594 (MultiSciences, 70-GAM5942, 1:200) or Alexa 555 (Invitrogen, A21422, 1:200).

### Cell lines used and cell culture

Stably transfected murine breast cancer cell line 4T1-ZsGreen1 (ZsGreen1 fluorescent protein, Excitation maximum: 493 nm, Emission maximum: 505 nm) and melanoma cell line B16F10-tdTomato (tdTomato fluorescent protein, Excitation maximum: 554 nm, Emission maximum: 581 nm), were obtained from Klink Biotechnologies (Shenzhen, China). 4T1-ZsGreen cells were maintained in RPMI 1640 with 10% fetal bovine serum (FBS) (PAA Laboratories) in a humidified atmosphere containing 5% CO_2_ at 37 °C. B16F10-tdTomato cells were grown in Dulbecco’s modified Eagle’s medium (DMEM; Gibco) with 10% fetal bovine serum and 1% penicillin/streptomycin (Gibco) and incubated at 37 °C in 5% CO_2_-humidified atmosphere.

### Tumor model

Female BALB/c and C57BL/6 mice (5–6 weeks, 18–22 g) were obtained from Guangdong province Laboratory Animal Center (Guangzhou, China). 4T1-ZsGreen1 cells (1.25 × 105) were suspended in 0.05 ml phosphate buffered saline (PBS) and then injected into the footpads of the BALB/c mice. B16F10-tdTomato (2.5 × 105) were suspended in 0.1 ml PBS and then injected subcutaneously into the backs of the mice. All experiments were performed in accordance with the Guide for the Care and Use of Laboratory Animals, with the approval of the Shenzhen University, Shenzhen, China.

### Quantification of fluorescence intensities of brain slices

1-mm-thick brain slices of Thy1-YFP-G mouse line were used to evaluate the stability of FPs in tissues for different clearing methods. The fixed brain slices were washed in PBS and cleared by various optical clearing agents including UbasM, CUBIC and uDISCO (incubation at room temperature 20 °C) and Sca*l*eSQ(5), Sca*l*eSQ(0) and SeeDB (incubation at 37 °C) and the control (PBS(-)). A Zeiss SteREO DiscoveryV12 equipped with a 1 × objective lens (WD = 81mm) with a cooled CCD camera was used to evaluate quenching of fluorescent proteins in the clearing agents. The brain slices were imaged over the same period of time under the same illumination. Relative FP preservation was calculated by the ratio of the total fluorescence intensity of the sample after clearing to the intensity before clearing. The total fluorescence intensity was represented by the summation of the grey values of all pixels in each sample. At least 3 different individual samples (different age and sex) were measured for each clearing method. Average FP preservation ratio and its standard deviations were included in Fig. 2. PBS as the control reagent exhibits ~100% FP preservation after 1 h, 2 h or even 24 h.

### Confocal microscopy

Multiple regions of various mouse samples were imaged with an inverted laser scanning confocal microscopy system (Zeiss, LSM710) with a 10 × objective lens (Plan-Neofluar, NA = 0.3, WD = 5.2 mm). The z step size was ~9 μm.

### OpenSPIM

Observation was performed with a home-built OpenSPIM system equipped with a 10 × objective lens (Olympus LMPLFLN, NA = 0.25, WD = 21 mm). Cleared agarose-embedded samples were positioned under a 4D stage in front of the objective in the mounting medium (Ub-2). The *z* step size was set 10 μm for samples (Fig. 4).

### OPT

400 wide-field 2D projection images were acquired by the CCD at 0.9° angular intervals over a full rotation (360°) of the sample though detection optics (OPTEM 35–41-10–000) and then reconstructed using a standard filtered back-projection algorithm. Cleared agarose-embedded samples were placed under the rotation stage in the mounting medium (Ub-2).

### Data availability

All data generated or analysed during this study are included in this published article (and its Supplementary Information files).

## Electronic supplementary material

Supplementary materials

3D imaging of YFP-expressing neurons in Cortex region acquired with CLSM

3D imaging of YFP-expressing neurons in Hippocampus region acquired with CLSM

3D reconstruction of a 2-mm-thick brain slice from a thy1-YFP (H-line) mouse acquired with OPT

3D reconstruction of a mouse lung sample showing metastatic spread of murine breast cancer cell line 4T1-ZsGreen1 from foot pad locations acquired with OPT

3D reconstruction of a mouse lung sample showing metastatic spread of melanoma cell line B16F10-tdTomato from subcutaneous locations acquired with OPT
