# Supplementary material for: UbasM: An effective balanced optical clearing method for intact biomedical imaging
Source: Sci Rep. 2017 Sep 22;7:12218. doi: 10.1038/s41598-017-12484-3 (PMC5610269; doi:10.1038/s41598-017-12484-3)
Supplement: Supplementary file 1 — Supplementary materials [file 41598_2017_12484_MOESM1_ESM.pdf]

UbasM: An effective balanced optical clearing method for intact biomedical imaging

Lingling Chen*[,1], Guiye Li[1], Yamin Li[1], Yingchao Li[1], Haiou Zhu[1], Li Tang[2], Paul French[3], James McGinty[3], and Shuangchen Ruan*[,1]

[1] College of Optoelectronics Engineering, Shenzhen University, Shenzhen, Guangdong, 518060, P. R. China.
[2] Department of Medicine, Shenzhen University, Shenzhen, Guangdong, 518060, P. R. China.
[3] Photonics Group, Department of Physics, Imperial College London, SW7 2AZ, UK.
* l.chen10@szu.edu.cn or scruan@szu.edu.cn

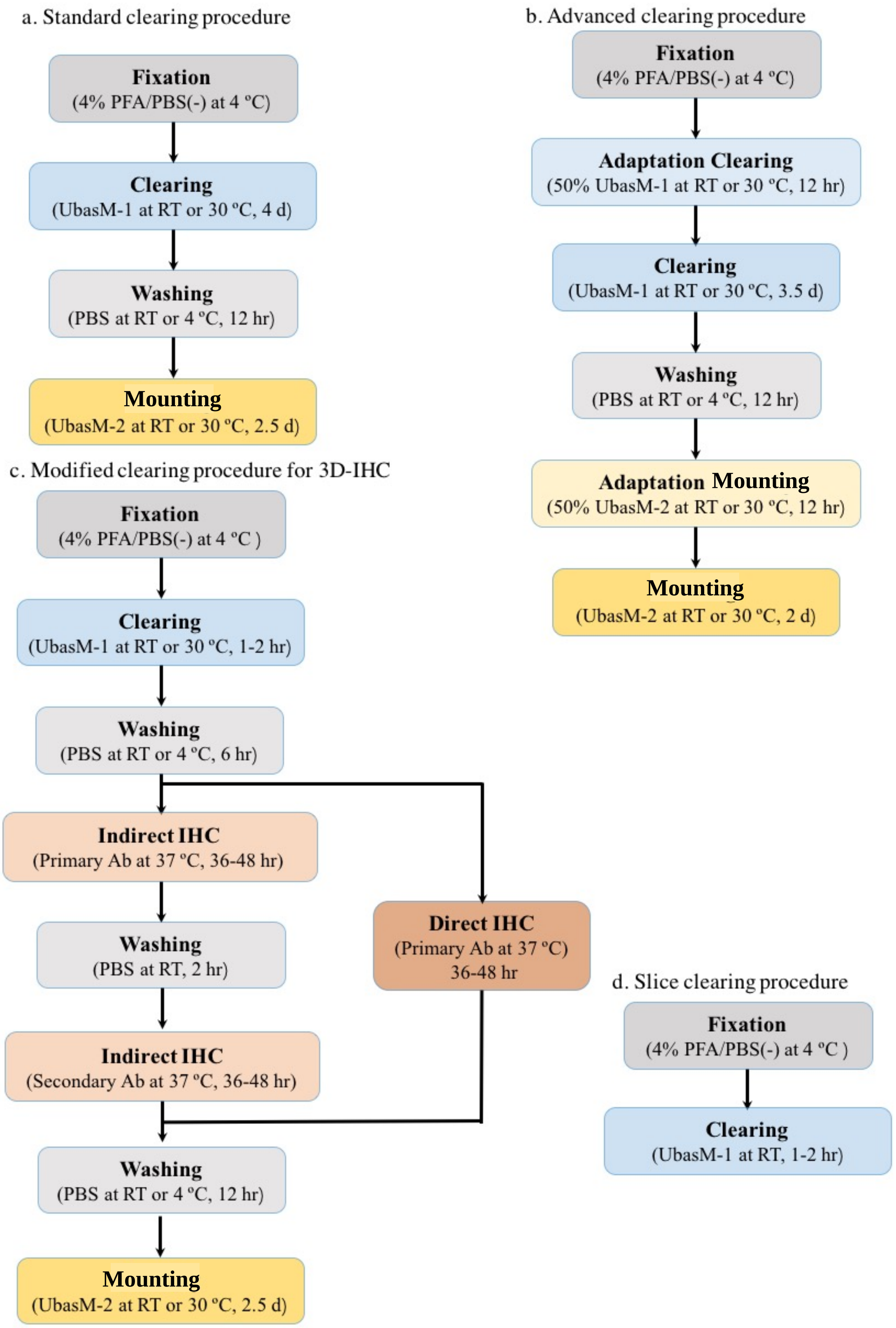

Supplementary Figure 1

Experimental protocols for standard, advanced, 3D-IHC and slice clearing UbasM

(a) Standard and (b) Advanced UbasM clearing protocols for adult mouse hemisphere (i.e. large tissue block). When thin or small tissue samples are used, the incubation time for each step can be substantially shortened. (c) Modified clearing protocol for 3D-IHC UbasM for ~1-2 mm thick brain slice. (d) Easy clearing procedure for mm thick slices.

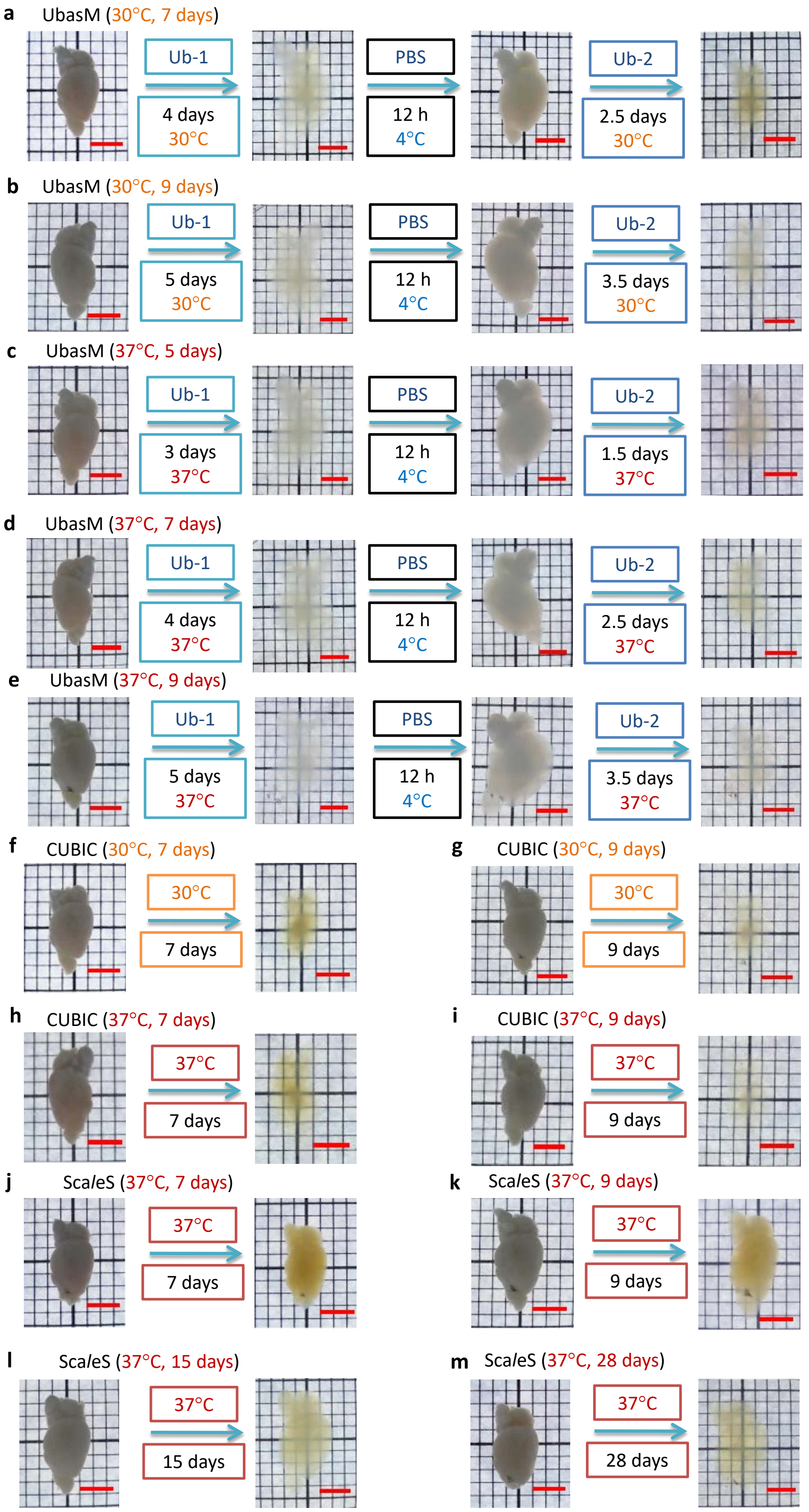

Supplementary Figure 2

Comparative observation of the clarification and clearing efficiency among mouse hemispheres treated by different reagents

4-week-old mouse hemisphere brain samples were cleared according to the protocols of UbasM, CUBIC and Sca*l*eS after fixation with 4% PFA. At time points indicated, clarification of the samples was examined by taking photos. (a-b) UbasM-treated hemispheres at 30 °C for 7 and 9 days; (c-e) UbasM-treated hemispheres at 37 °C for 5, 7 and 9 days; (f-g) CUBIC-treated hemispheres at 30 °C for 7 and 9 days; (h-i) CUBIC -treated hemispheres at 37 °C for 7 and 9 days; (j-m) Sca*l*eS-treated hemispheres at 37 °C for 7, 9, 15 and 28 days. Scale bar: 5 mm.

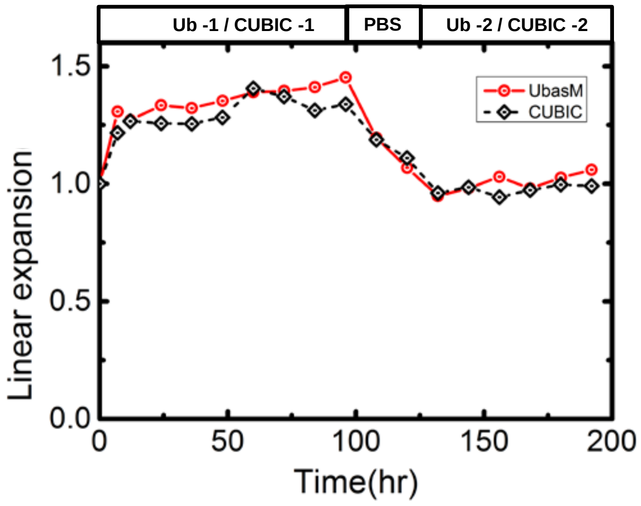

Supplementary Figure 3

Sample expansion and shrinkage during the optical clearing

Hemisphere samples (4-week-old) were cleared with UbasM and CUIBIC for 7 days according to the timeline shown at the bottom. Optical clearing using Ub-1 or CUBIC-1 caused ~150% sample linear expansion at first 96 hours. After PBS and Ub-2 or CUBIC-2 phase, samples were shrunk to ~100%.

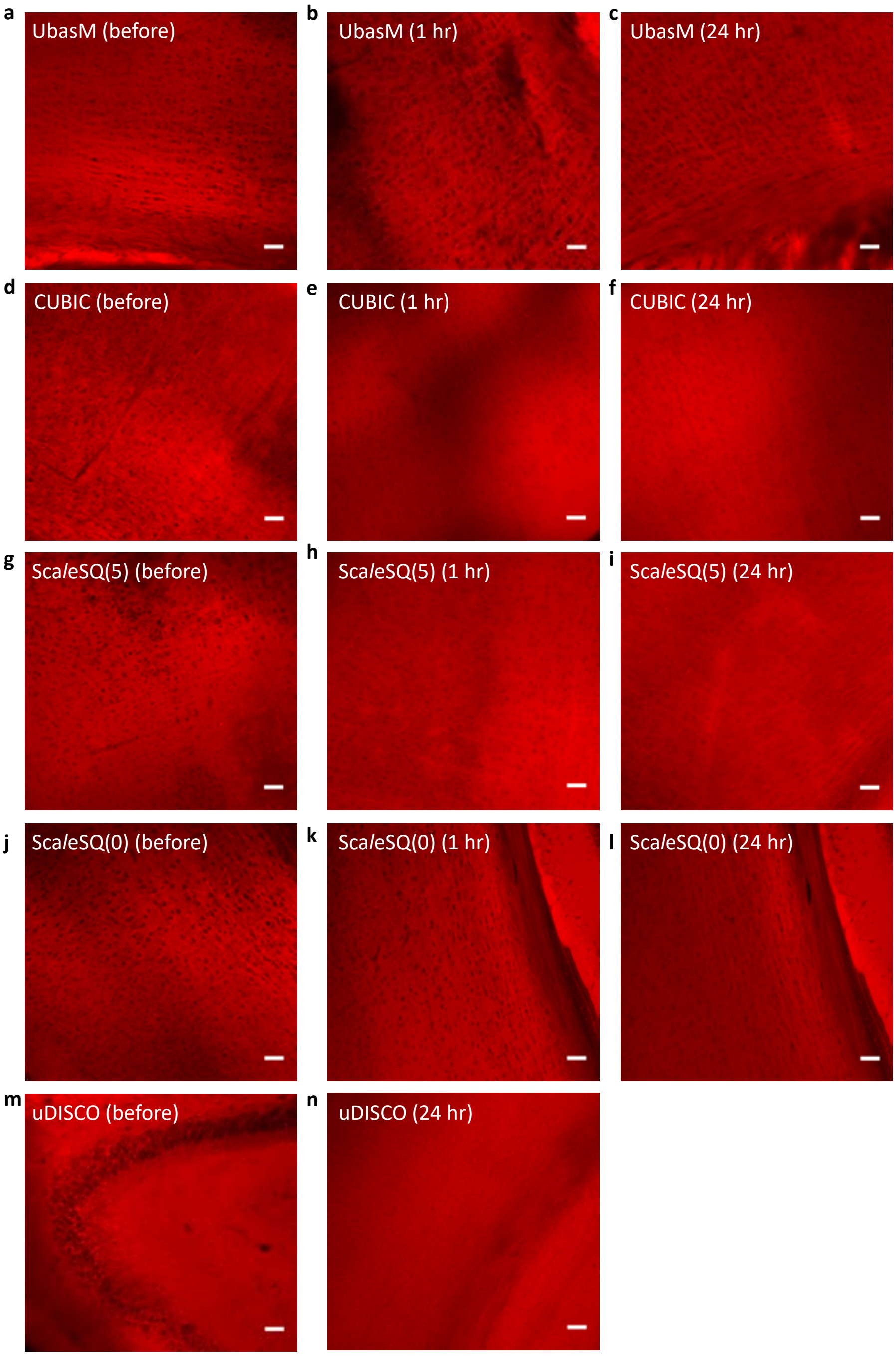

Supplementary Figure 4

Comparative observation of the DiI labeling compatibility with different clearing reagents

DiI-labeled neural tracts in the 12-week-old female mouse brain samples were well preserve with clarification by UbasM and Sca*le*SQ(0) but not by CUBIC, Sca*le*SQ(5) and uDISCO. left, fluorescence images showing DiI labeling before clarification. middle and right, images showing DiI-labeled neuritis in the cleared sample treated by different reagents after 1 hr and 24 hr. The sample was imaged using a confocal microscope (Zeiss, LSM 710). Scale bar: 50 µm.

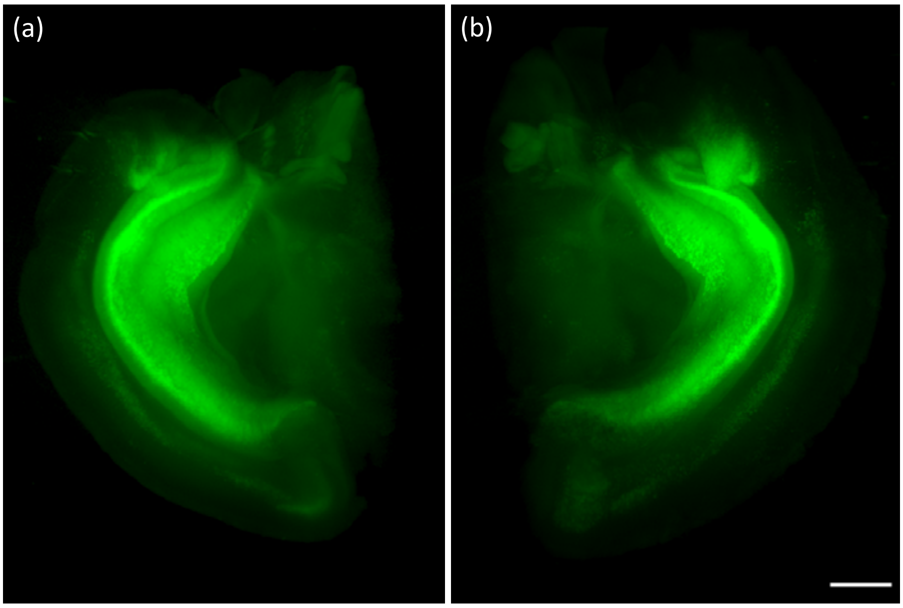

Supplementary Figure 5

3D reconstruction of 4-mm-thick mouse brain slice cleared by UbasM

Reconstructed (maximum intensity projection) images of 4-mm-thick thy1-YFP (H-line) mouse brain slice (10-week-old female) acquired with OPT from two directions. Scale bar: 1 mm.

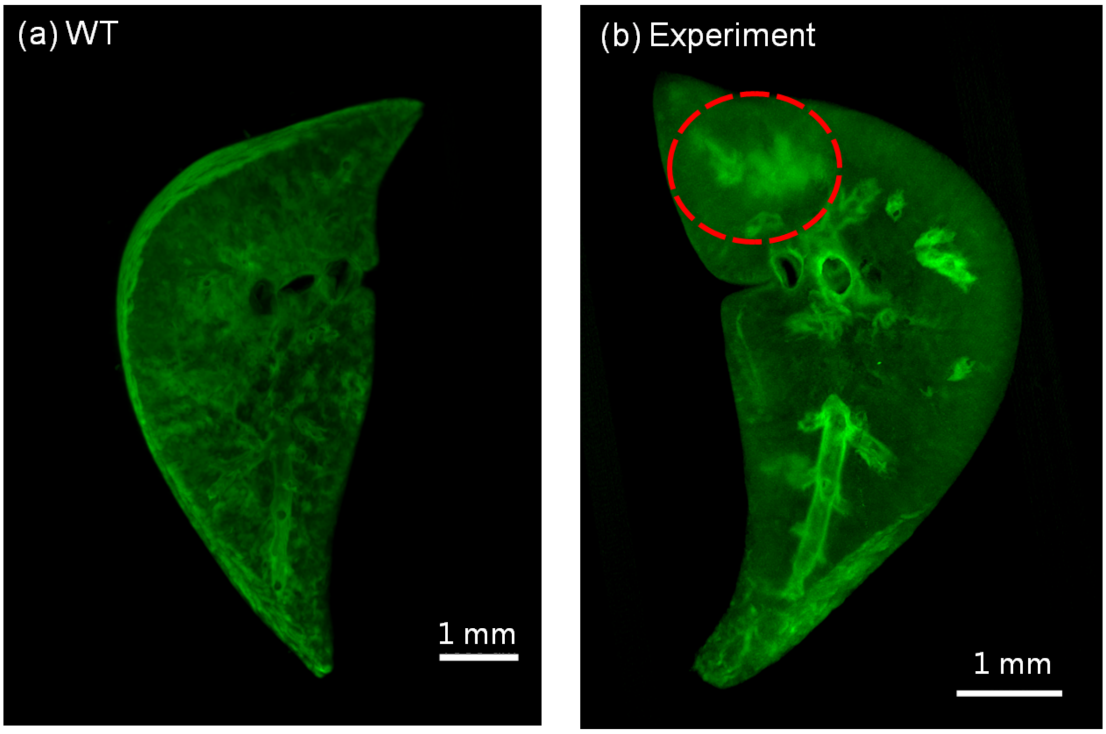

Supplementary Figure 6

Comparison of UbasM-treated tissue samples between the control and metastasis of murine breast cancer cell line 4T1-ZsGreen1

Samples showing 3D imaging of (a) the control lung sample (wild type - WT) and (b) the experiment sample from metastasis of murine breast cancer cell line 4T1-ZsGreen1 (from foot pad locations) acquired with OPT. The red circle in (b) indicates the clusters of metastasized cells. Scale bars represent 1 mm.

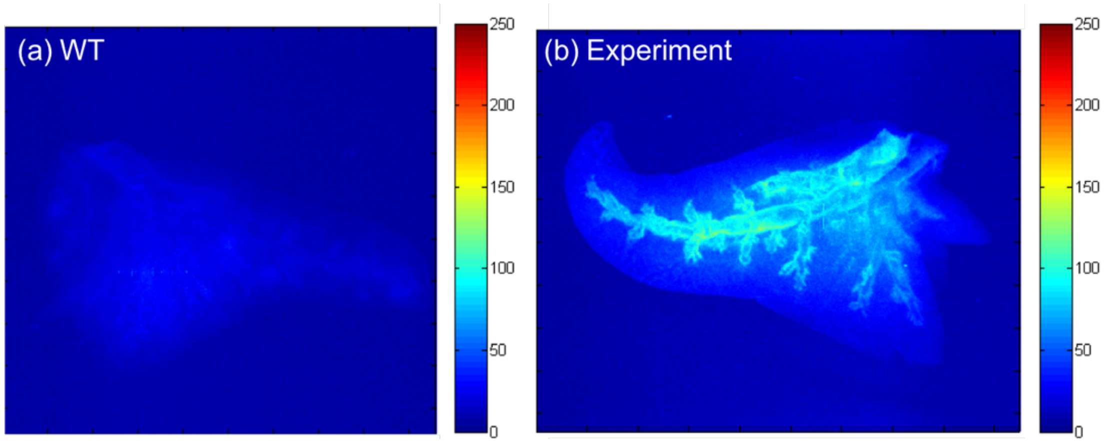

Supplementary Figure 7

Comparison of UbasM-treated tissue samples between the control and metastasis of melanoma cell line B16F10-tdTomato

Samples showing 3D imaging of (a) the control lung sample (wild type - WT) and (b) the experiment sample from metastasis of melanoma cell line B16F10-tdTomato from subcutaneous locations acquired with OPT at the same intensity scale to demonstrate the difference. It is noted that there is substantially higher fluorescence of metastatic cancer cells within the bronchi of the lung in the Experiment mouse as compared to the WT mouse. This bright fluorescence within the bronchi may indicate how cancer cells spread from subcutaneous locations to the lungs.

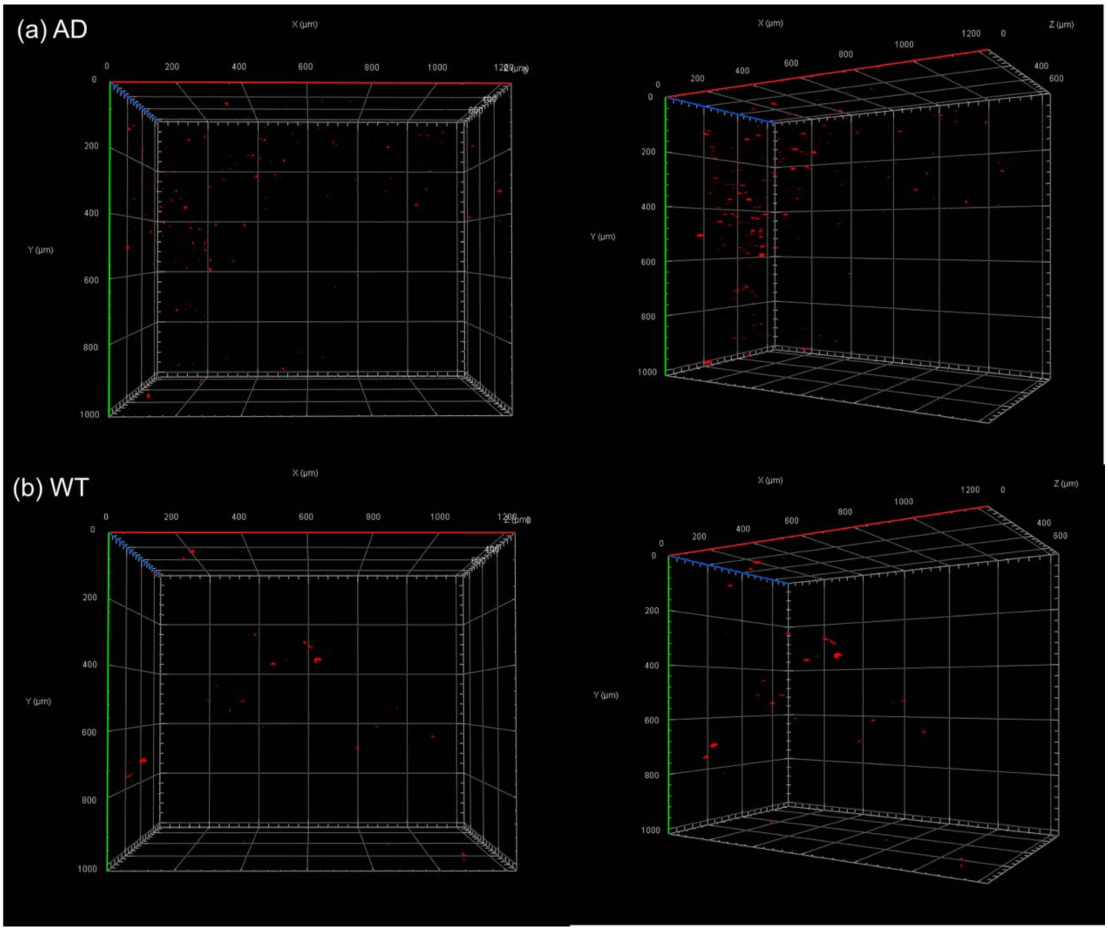

Supplementary Figure 8

3D imaging of Aβ plaques in similar subareas of the brain after UbasM clarification

3D imaging in two exemplary samples: (a) one AD mouse model brain slice and (b) the control (one WT mouse brain slice) by 3D-IHC with DyLight-594 (red) from two views, illustrating how Aβ plaques (red spots) inside a 1-mm-thick slice were immunostained. The images presented here were imaged in similar sub-areas of the brain (in hippocampus region) in both samples using confocal microscopy. 15-month-old mice are used. 238 and 34 plaques were identified inside the areas shown in (a) AD and (b) WT brain samples respectively. AD – Alzheimer's disease; WT – wild type.

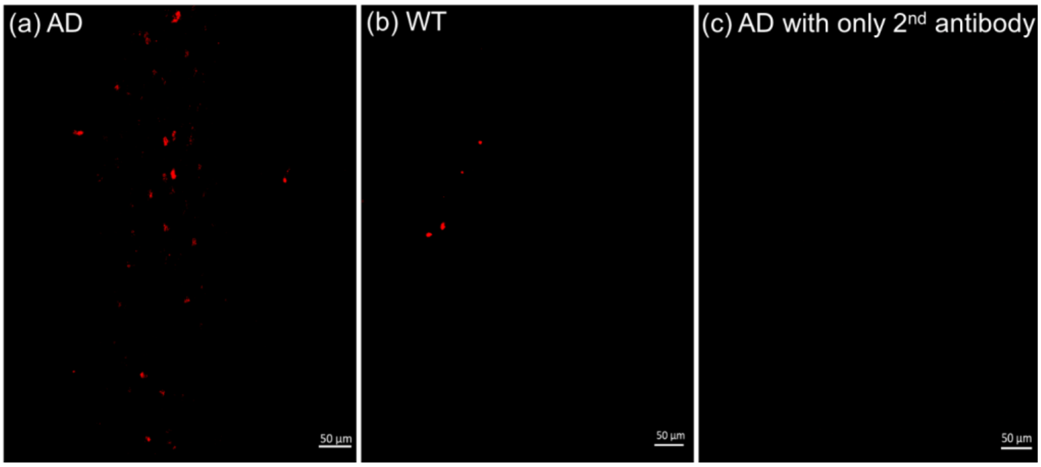

Supplementary Figure 9

Imaging of Aβ plaques in the brain slices after UbasM clarification

Confocal imaging in (a) AD mouse model brain slice stained with primary and secondary antibodies; (b) the WT mouse brain slice stained with primary and secondary antibodies as control and (c) AD mouse model brain slice stained with only secondary antibodies as control to rule out the possibility of secondary antibody aggregation. They illustrate how Aβ plaques (red spots) inside brain slices were immunostained. 12-month-old mice are used in this experiment. AD – Alzheimer's disease; WT – wild type. Scale bar, 50 µm.
